# Longitudinal associations between the infant gut microbiome and negative affect in toddlerhood

**DOI:** 10.1017/S0954579425100229

**Published:** 2025-06-05

**Authors:** Sarah C. Vogel, Nicolas Murgueitio, Nicole Huth, Kathy Sem, Rebecca C. Knickmeyer, Sarah J. Short, Roger Mills-Koonce, Cathi Propper, Nicholas J. Wagner

**Affiliations:** 1 Department of Psychological and Brain Sciences, Boston University, Boston, MA, USA; 2 Department of Psychology and Neuroscience, University of North Carolina at Chapel Hill, Chapel Hill, NC, USA; 3 Department of Pediatrics and Human Development, Michigan State University, East Lansing, MI, USA; 4 Institute for Quantitative Health Science and Engineering, Michigan State University, East Lansing, MI, USA; 5 Department of Educational Psychology, University of Wisconsin-Madison, Madison, WI, USA; 6 Center for Healthy Minds, University of Wisconsin-Madison, Madison, WI, USA; 7 School of Education, University of North Carolina at Chapel Hill, Chapel Hill, NC, USA; 8 School of Nursing, University of North Carolina at Chapel Hill, Chapel Hill, NC, USA; 9 Frank Porter Graham Child Development Institute, University of North Carolina at Chapel Hill, Chapel Hill, NC, USA

**Keywords:** Gut microbiome, infancy, microbiome-gut-brain axis, negative affect, sensitive periods

## Abstract

The role of the gut microbiome in infant development has gained increasing interest in recent years. Most research on this topic has focused on the first three to four years of life because this is a critical period for developing gut-brain connections. Prior studies have identified associations between the composition and diversity of the gut microbiome in infancy and markers of temperament, including negative affect. However, the specific microbes affected, and the directionality of these associations have differed between studies, likely due to differences in the developmental period of focus and assessment approaches. In the current preregistered study, we examined connections between the gut microbiome, assessed at two time points in infancy (2 weeks and 18 months), and negative affect measured at 30 months of age in a longitudinal study of infants and their caregivers. We found that infants with higher gut microbiome diversity at 2 weeks showed more observed negative affect during a study visit at 30 months. We also found evidence for associations between specific genera of bacteria in infancy and negative affect. These results suggest associations between specific features of the gut microbiome and child behavior may differ based on timing of gut microbiome measurement.

## Introduction

In recent years, there has been considerable growth in research examining the role of the gut microbiome in health and development. In adults,accumulating research has implicated the gut microbiome in several different forms of psychopathology (Butler et al., 2023; Jiang et al., 2015; Li et al., 2021), and research in children suggests that differences in the composition and diversity of the gut microbiome as early as infancy may have implications for mental health throughout the lifespan (Vogel et al., 2020). Several existing studies have identified associations between the gut microbiome and multiple temperamental indicators of risk for later psychopathology in infancy and early childhood, including fear behaviors (Carlson et al., 2021) and negative affect (Aatsinki et al., 2019; Huovinen et al., 2024). However, inconsistencies in the strength and direction of these associations, in part due to a dearth of longitudinal studies in this area, have limited our ability to draw specific conclusions about whether and how the gut-brain-behavior connections contribute to patterns of adaptation across development. Here, we add to this growing body of literature by examining longitudinal associations between markers of gut microbiome assessed at two time points during infancy (2 weeks and 18 months old) and behavioral measures of negative affect at 30 months (approximately 2.5 years of age).

A greater tendency towards negative affect in early life is associated with elevated risk for internalizing and externalizing problems in childhood (Affrunti & Woodruff-Borden, 2016; Calkins & Fox, 2002; Compas et al., 2004; Edwards & Hans, 2015). Likewise, an emerging body of research establishes associations between the composition and diversity of the infant gut microbiome and the development of psychopathology (McKenna et al., 2023; Querdasi, Enders, et al., 2023). Research characterizing early temperamental risk for psychopathology has been transformative in our ability to develop targeted intervention and prevention strategies, which are thought to be more effective earlier in development (Bayer et al., 2011; Chronis-Tuscano et al., 2022). As such, enhancing our scientific understanding of how early differences in the composition and functioning of the gut microbiome in infancy predict negative affect offers additional potential for prevention and intervention to promote healthy emotional development.

The gut microbiome’s influence on individual differences in negative affect has gained increasing interest in the literature. Experimental studies in rodents have provided causal evidence that disruptions to the gut microbiome are associated with altered emotional functioning, including changes in fear extinction and expression, and models of anxiety and depression (Bravo et al., 2011; Cowan et al., 2019; Heijtz et al., 2011). Across studies, rodents bred without gut microbiota have shown deficits in social and emotional behavior and displayed differences in anxiety- and depression-like behaviors (Campos et al., 2016; Heijtz et al., 2011; Hoban et al., 2018; Lukić et al., 2019; Luo et al., 2018; Sudo et al., 2004; Swartz et al., 2012). However, studies in rodents have found that supplementation of the gut microbiome with strains of *Lactobacillus* (Cowan et al., 2016, 2019) and *Bifidobacterium* (Luk et al., 2018), two early colonizers of the human infant gut microbiome with well-documented anti-inflammatory properties, can reverse stress-induced changes in cognition and behavior, but these effects are unique to infancy (Sudo et al., 2004). This body of research provides causal evidence for connections between the infant gut microbiome and behavior and points towards specific taxa, namely *Lactobacillus* and *Bifidobacterium*, as potential candidates for additional study in humans.

In humans, there is growing evidence for associations between the diversity and composition of the infant gut microbiome and temperament throughout early childhood. Previous studies have identified links between several generaof bacteria and affect across infancy and toddlerhood, including *Bifidobacterium* (Aatsinki et al., 2019; Fan et al., 2023; Fox et al., 2021), *Bacteroides* (Aatsinki et al., 2019; Carlson et al., 2021), *Veillonella* (Carlson et al., 2021), and *Streptococcus* (Aatsinki et al., 2019), among others. Likewise, several studies have identified associations between gut microbiome alpha diversity, a global measure of the variety of microbial taxa present in the gut, and negative reactivity (Aatsinki et al., 2019; Fan et al., 2023), fear behaviors (Aatsinki et al., 2019; Carlson et al., 2021; Huovinen et al., 2024), and emotionality (Aatsinki et al., 2019; Fan et al., 2023) in infancy and toddlerhood. Importantly, however, the directionality of associations between alpha diversity and affect have been mixed, with some studies finding higher diversity associated with more positive affect (Christian et al., 2015) and some with more negative affect (Aatsinki et al., 2019; Carlson et al., 2021; Huovinen et al., 2024), often differing based on when in development the gut microbiome was measured (Carlson et al., 2021; Fan et al., 2023). Work in humans has also found that higher alpha diversity at one month of age is associated with greater fear reactivity at 12 months (Carlson et al., 2021), and that higher alpha diversity at 12 months is associated with decreased resting functional connectivity between the amygdala and thalamus, brain regions that support emotional development (Gao et al., 2019). Greater alpha diversity in infancy is thought to reflect an accelerated maturation of the gut microbiome, suggesting that higher diversity in infancy may not confer the same benefits in the infant gut as it does in adults (Stewart et al., 2018).This body of research highlights the need for longitudinal studies of the gut microbiome and child development that measure the gut microbiome at multiple points throughout infancy and early childhood to better characterize early microbiome-gut-brain associations.

The gut microbiome undergoes considerable change in the first 3–4 years of life, which is a critical period in the system of connections between the gut microbiome and the brain, known as the microbiome-gut-brain axis (Callaghan, 2020; Cowan et al., 2020). During this time, the systems that make up the microbiome-gut-brain axis undergo overlapping periods of sensitivity to environmental influence, making questions about the influence of the gut microbiome on children’s temperament essential to consider from a developmental perspective (Callaghan, 2020). Longitudinal studies incorporating in-depth measurement of the gut microbiome across infancy have found evidence for distinct phases of gut microbiome maturation across the first 3 years of life (Beller et al., 2021; Stewart et al., 2018), suggesting that the influences of the gut microbiome on brain and behavioral development may differ based on developmental timing even within this sensitive period. These developmental characteristics of the infant gut, coupled with the mixed directionality of existing research on infant gut microbiome and temperament, emphasizes the need for longitudinal research incorporating gut microbiome measurement at multiple points throughout infancy.

### The current study

The current study examined longitudinal associations between gut microbiome diversity and composition at 2 weeks and 18 months of age and behavioral measures of negative affect at 30 months old. Given the previous findings discussed above, we hypothesized that higher gut microbiome alpha diversity at both time points would be associated with more negative affect in toddlerhood but that greater abundances of bacteria from the genera *Bifidobacterium, Lactobacillus,* and *Bacteroides* across infancy would be associated with less negative affect when children were 30 months old. We hypothesized that additional genera of bacteria would be associated with negative affect, which we tested in exploratory analyses, and that variation in between-subjects diversity (beta diversity) would be associated with variation in negative affect. Hypotheses and analytic procedures were preregistered with the Open Science Framework (OSF) as part of a larger project at: https://osf.io/9ynqe.

## Methods

### Participants and procedures

Data for this study came from a longitudinal study which recruited 203 pregnant women during the second trimester of their pregnancy. Participants were recruited from counties surrounding the University of North Carolina Chapel Hill. Pregnant women were identified via electronic medical records from the university hospital, as well as via online advertising, community flyering and in-person communication at local WIC clinics. Inclusion criteria included: singleton pregnancy, maternal age≥18 years, English fluency, and residing within the target recruitment area with no plans to move out of the area in the next 3 years. Participants who were recruited but were unable to come to the lab for a prenatal data collection visit prior to 37 weeks gestation were also excluded, as were participants who miscarried or terminated their pregnancies. Finally, infants were excluded after birth if they were born with a low birthweight (< 2500g); born with a gestational age shorter than 36 weeks, 4 days; stayed in the NICU >24 hours after birth; were diagnosed with a chronic medical condition at birth; received surgery immediately after birth; had metal devices implanted into the body (e.g., cochlear implants); or if the family was unreachable or otherwise unwilling to participate in the 2-week study visit. Additional information about recruitment and study design has been previously published (Mills-Koonce et al., 2022).

Upon study enrollment, expectant caregivers completed several questionnaires about demographics and household composition. When infants were two weeks old and again at 18 months, families were provided a biospecimen collection kit with instructions and all necessary materials to collect a stool sample from an infant diaper, including two tubes filled with 1 ml All protect reagent (Valencia, CA), which preserves samples up to seven days at room temperature. Caregivers were instructed to collect a small sample of infant stool (~200 mg) from a diaper, place the sample in the tube with the reagent, and bring the sample to a lab visit. Samples were then stored at − 80 °C until sequencing. For the 30-month study visit, families were mailed a standardized set of materials for a virtual visit from their home, during which families completed several activities over Zoom with a researcher present. 96 children (47% female) provided a stool sample for at least one time point *and* completed a virtual study visit at 30 months. 76 children provided a stool sample at 2 weeks and had complete behavioral data from 30 months; 69 children provided a stool sample at 18 months and had complete behavioral data from 30 months. There were no significant differences between the 2-week and 18-month samples based on SES (*t* = −0.62, *p* = .54), breastfeeding duration (*t* = −0.26, *p* = .79), infant sex (*X*^*2*^ = 0, *p* = 1), infant race (*X*^*2*^ = 1.59, *p* = .81), or method of delivery (*X*^*2*^ = 0, *p* = 1). Sample characteristics are summarized in Table 1.

### Measures

#### Infant gut microbiome diversity and composition

The gut microbiome measures of interest in this analysis are alpha diversity, measured via the Shannon and Chao1 indices, beta diversity measured with Bray-Curtis distance, and relative abundance of species and genera in samples. The Shannon index reflects both the number of distinct bacterial groups (richness), and distribution of those members (evenness). A higher value of Shannon’s diversity suggests that a sample has a larger number of bacterial groups with relatively similar number of members of those groups. The Chao1 index accounts for the number of observed species, the number of singletons (species captured once), and the number of doubletons (species captures twice). High Chao1 diversity scores reflect many bacterial groups but also reflect a situation where most species are only observed once or twice per sample. Bray-Curtis distance is a between-subjects measure reflecting similarity/dissimilarity between two microbial communities that accounts for both the presence and abundance of bacterial groups. Relative abundances reflect the proportion of organisms belonging to a specific taxon of interest in relation to the total number of organisms identified.

#### DNA isolation.

Fecal samples were prepared using ZymoBIOMICS^™^ Spike-in Control I (High Microbial Load) (Cat#: D6320). 10 mg of spiked fecal sample was then transferred to a 2 ml tube containing 200 mg of 106/500 μm glass beads (Sigma, St Louis, MO) and 0.5 ml of Qiagen PM1 buffer supplemented with 600IU of Qiagen proteinase K (Valencia, CA) followed by a 70°C incubation for 1h. Mechanical lysis was performed for 20 minutes on a Digital Vortex Mixer. After a 5-minute centrifugation, 0.45 ml of supernatants was aspirated and transferred to a new tube containing 0.15 ml of Qiagen IRS solution. The suspension was incubated at 4°C overnight. After a brief centrifugation, supernatant was aspirated and transferred to deep well plates containing 0.45 ml of Qiagen binding buffer supplemented with Qiagen ClearMag Beads. DNA was purified using the automated KingFisher^™^ Flex Purification System and eluted in DNase free water (Allali et al., 2017; Azcarate-Peril et al., 2018; Guadamuro et al., 2019).

#### Illumina whole genome shotgun sequencing.

5 ng of genomic DNA was processed using the Nextera XT DNA Sample Preparation Kit (Illumina). Target DNA was simultaneously fragmented and tagged using the Nextera Enzyme Mix containing transposome that fragments the input DNA and adds the bridge PCR (bPCR)-compatible adaptors required for binding and clustering in the flow cell. Next, fragmented and tagged DNA was amplified using a limited-cycle PCR program. In this step index 1 (i7) and index 2 (i5) was added between the downstream bPCR adaptor and the core sequencing library adaptor, as well primer sequences required for cluster formation. The thermal profile for the amplification had an initial extension step at 72°C for 3 min and initial denaturing step at 95°C for 30 sec, followed by 15 cycles of denaturing of 95°C for 10 s, annealing at 55°C for 30 s, a 30 s extension at 72°C, and final extension for 5 minutes at 72°C. The DNA library was then be purified using Agencourt^®^ AMPure^®^ XP Reagent. Each sample was quantified and normalized prior to pooling. The DNA library pool was loaded on the Illumina platform reagent cartridge (Illumina) and on the Illumina instrument (Seth-Smith et al., 2019). Sequencing yielded a median of 8,382,776 reads [range 1,073,246–17,058,056] at 2 weeks and a median of 8,197,095 reads [range 2,892,855–15,777,647] at 18 months after quality control.

#### Classification and abundances.

Sequencing output from the Illumina MiSeq/HiSeq/4000 platform were converted to fastq format and demultiplexed using Illumina Bcl2Fastq 2.20.0 (Illumina, Inc., 2018). Quality control of the demultiplexed sequencing reads was verified by FastQC (Babraham Institute, 2014).Adapters were trimmed using Trim Galore (Babraham Institute, 2017). The resulting paired-end reads were classified with Kraken2 and Bracken 2.5 (Lu et al., 2017; Wood et al., 2019) and all reads identified as host were eliminated. Paired-end reads were joined with vsearch 1.10.2 (Rognes et al., 2016). The resulting single-end reads were again trimmed of any remaining adapters using Trim Galore. Estimates of taxonomic composition, gene family, path abundance, and path coverage were produced from the remaining reads using HUMAnN2 (Abubucker et al., 2012). Results were visualized using QIIME 2 (Caporaso et al., 2010). Cell counts were estimated from an assumed 20 million *Imtechella halotolerans* cells as per manufacturer’s instructions.

#### Toddler negative affect

Families participated in a virtual study visit when the child was 30 months old and completed study tasks from their home including a parent-child interaction task, several questionnaires, and biospecimen collections with a researcher present over Zoom. The study visit lasted approximately 1.5 hours. After the study visit, the researcher completed a modified version of the Infant Behavior Record (IBR), a global behavior rating scale form that assesses child behaviors across the course of the study visit (Bayley, 1969).The first two items, related to responsiveness to observers and other persons present, were excluded because they did not apply to a Zoom visit. The IBR was completed by a single rater. Here, we focus on IBR items pertaining to degree of happiness, reaction to the new or strange, and degree of irritability across the study visit. Behaviors are rated on a 9-point scale, where 1 is the lowest value (indicating the behavior was rarely or not observed) and 9 is the highest value (indicating that the behavior was observed the whole visit). The degree of happiness item lists a 1 as “Child seems unhappy throughout the home visit,” a5 as “Moderately happy or contented; may become upset, but recovers fairly easily,” and a 9 as “Radiates happiness; nothing is upsetting; animated.” The reaction to new/strange item lists a 1 as “Accepts the entire situation with no evidence of fear, caution, or inhibition of actions,” a score of 5 reads “Behavior is affected by the new and strange, but just moderately and for approximately the first third of the home visit,” and a score of 9 indicates “Strong indication of fear of the strange, to the extent that he cannot be brought to play or respond to the examiner or tasks.” Finally, a score of 1 on the irritability item indicates “No irritability; infant responds passively to all stimulation,” a score of 5 reads “Irritability to aversive and non-aversive stimulation leads to high intensity crying, but with consoling returns to lower states,” and a score of 9 indicates “Irritable to all degrees of stimulation encountered throughout the home visit.” We used structural equation modeling to create a latent variable of negative affect using the *lavaan* package in RStudio (R Core Team, 2023; Rosseel, 2012), consisting of the three IBR items described above. After fitting the latent variable, we extracted factor scores using the lavPredict function and used them for further analyses. This factor score was the outcome of interest in all subsequent analyses. The negative affect latent variable measurement model was saturated, indicating perfect fit (CFI = 1.00, TLI = 1.00, SRMR = 0, RMSEA = 0) and all indicators loaded significantly onto the latent variable (Irritability: *λ* = 0.68; Happiness: *λ* = −0.89; Reactions to new/strange: *λ* = 0.51). The Cronbach’s alpha for the IBR as a whole was 0.75, and for the group of items used in our latent variable was 0.73.

#### Covariates

Caregivers reported on family social demographic characteristics, including caregiver education and family income, at study enrollment. We calculated a measure of family socioeconomic status (SES) as a composite of caregiver education and family income-to-needs ratio. Caregivers reported the child’s sex, race, method of delivery, and whether or not the infant was breastfeeding at two weeks of age on the Infant Health Questionnaire and the Feeding Practices Questionnaire. When infants were 18 months old, caregivers reported on whether the child was breastfeeding and amount of time (in months) the child had breastfed for. We controlled for the following variables in all regressions: child sex, child race, family SES, method of delivery, and breastfeeding. We included child sex given sex-based differences in associations between the gut microbiome and temperament in previous studies (Huovinen et al., 2024). We controlled for child race and family SES to account for demographic differences in early gut microbiome composition found in previous studies (Lewis et al., 2021; Mallott et al., 2023). Finally, we controlled for method of delivery and breastfeeding given that these are two well-documented influences on the gut microbiome in infancy (Dominguez-Bello et al., 2010; Stewart et al., 2018). In the 2-week models, we controlled for whether the infant was breastfeeding or not at 2 weeks of age, and in the 18-month models we controlled for months of breastfeeding reported by the caregiver at 18 months. If the child was still breastfeeding at 18 months, they were assigned a value corresponding to their exact age in months at the time of stool sample collection.

### Statistical analyses

#### Descriptive statistics

We performed a series of t-tests, correlations, and ANOVAs, based on variable type, to examine descriptive associations between demographic characteristics, gut microbiome alpha diversity, and negative affect.

#### Alpha diversity analyses

To test our hypothesis that higher alpha diversity across infancy would be associated with greater negative affect, we performed a series of correlations and regressions between the Shannon and Chao1 indices from both 2 weeks and 18 months of age and our measure of negative affect at 30 months. Regressions modeled alpha diversity indices separately from both time points and controlled for child sex, child race, family SES, method of delivery, and breastfeeding. We also ran regression models with both alpha diversity time points predicting negative affect and controlling for covariates.

#### Beta diversity analyses

To test our hypothesis that variation in between-subjects diversity would be associated with variation in negative affect, we first calculated Bray-Curtis distances from genus-level relative abundance data. Next, we performed a principal component analysis on Bray-Curtis distances to reduce dimensionality, then we performed correlations between the resulting principal coordinates and our measure of negative affect, and regressions controlling for covariates described above. Finally, we performed regressions with both Bray-Curtis principal coordinates in the model and controlling for child sex, race, family SES, method of delivery, and breastfeeding. We performed these analyses separately with data from 2-weeks and 18-months.

#### Differential abundance analyses

##### Hypothesis-driven.

To test our preregistered hypotheses that *Bifidobacterium*, *Lactobacillus*, and *Bacteroides* abundances would be negatively associated with negative affect, we first performed a series of bivariate correlations between relative abundance of each taxon of interest at each time point and our measure of negative affect (i.e. correlation between 2-week *Bifidobacterium* abundance and negative affect, correlation between 18-month *Bifidobacterium* abundance and negative affect, etc). Next, we performed a series of regressions, one for each taxon of interest from each time point, predicting negative affect and controlling for child sex, race, family SES, method of delivery, and breastfeeding. We log-transformed relative abundance values that were highly skewed (skew >2) for use in correlations and regressions.

##### Exploratory.

We performed Microbiome Multivariable Associations with Linear Models v2 (MaAslin2; Mallick et al., 2021) models on genus- and species-level microbial abundance data and separately at 2 weeks and 18 months to identify microbial taxa and from each time point that were significantly associated with variation in negative affect at 30 months. We specified default model type, transformation, and normalization parameters for all MaAslin2 models. We filtered our datasets to include only taxa that were present in at least 50% of samples. P-values were adjusted for multiple comparisons using the Benjamini-Hochberg procedure, with a threshold for significance of *q* < .25, as is commonly used in biomarker discovery approaches (Kelsey et al., 2020; Querdasi, Vogel, et al., 2023). A *q*-value of .25 indicates a potential false discovery rate of 25%, in other words that there is a 25% chance that the association identified is significant by chance instead of a true effect. After identifying taxa significantly associated with negative affect, we performed regressions controlling for child sex, race, family SES, method of delivery, and breastfeeding. We log-transformed highly skewed (skew >2) abundance measures for use in regressions.

#### Multiple comparisons corrections

After performing all regressions, we used the Benjamini-Hochberg procedure for calculating false discovery rate (FDR) corrected *q*-values, with a significance threshold of *q* < .05. We report these FDR-corrected q-values, along with confidence intervals, for all regressions.

## Results

### Descriptive statistics

Our measures of alpha diversity, the Shannon and Chao1 indices, were significantly positively correlated with one another at 2 weeks (*r* = .73, *p* < .001) and 18 months (*r* = .57, *p* < .001) of age. Shannon diversity values from 2 weeks and 18 months were not correlated with one another (*r* = .01, *p* = .90), but there was a positive correlation between 2-week and 18-month Chao 1 diversity (*r* = .31, *p* = .03). Both measures of alpha diversity were significantly higher at 18 months than at 2 weeks (Shannon: *t* = −16.23, *p* < .001; Chao1: *t* = −16.51, *p* < .001). Negative affect at 30 months was not correlated with family SES (*r* = .01, *p* = .91) or exact age in months at the 30-month visit (*r* = −.06, *p* = .54). Average negative affect did not differ based on child sex (*t* = −.21, *p* = .83), method of delivery (*t* = −.71,*p* = .49), or child race (*F* = .18, *p* = .68).

### Alpha diversity analyses

**Figure 1.**
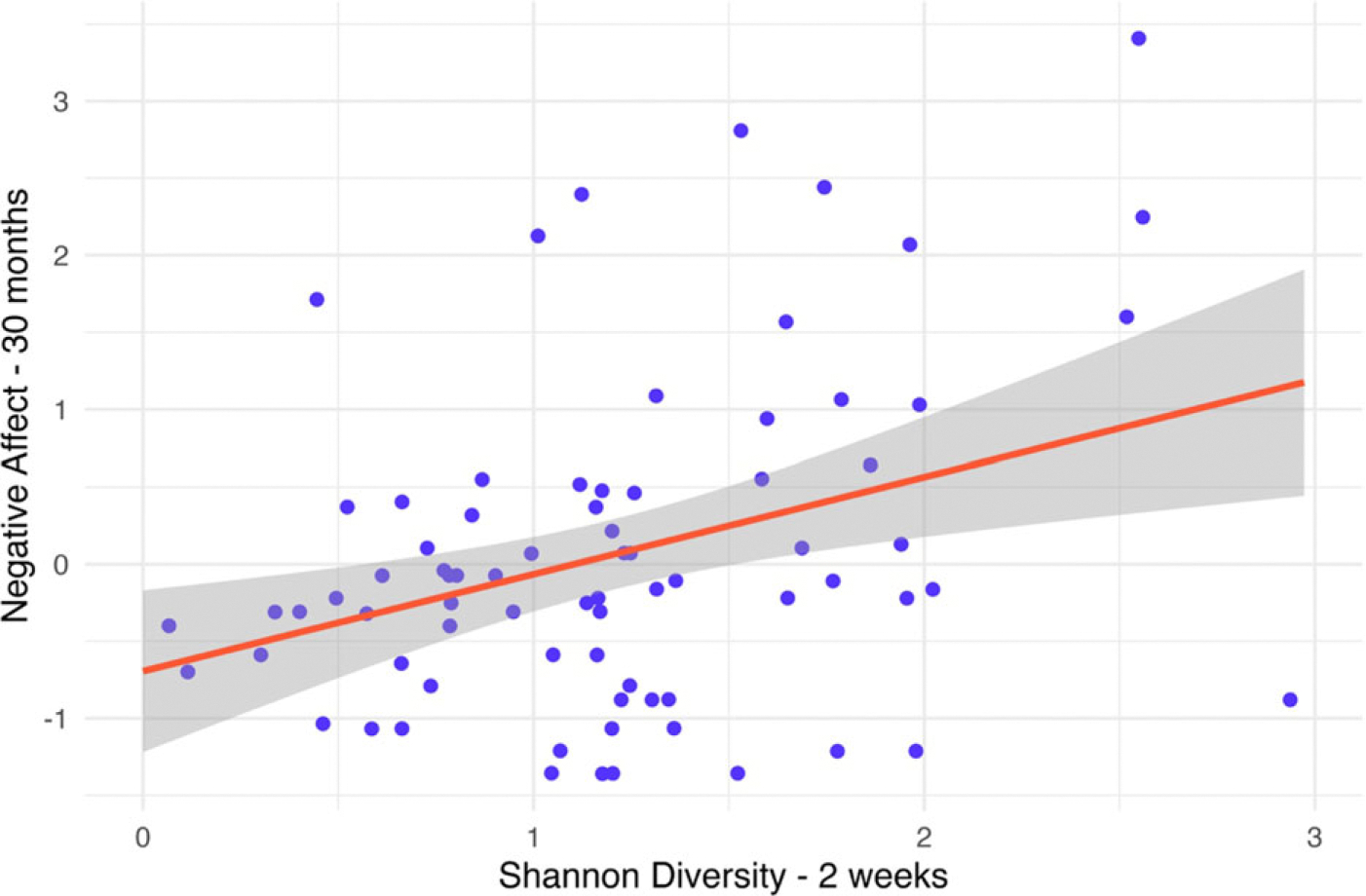
Positive associations between Shannon diversity at 2 weeks of age and negative affect at 30 months of age. Gray shading represents a 95% confidence interval, and points are jittered along the *x*-axis for ease of visibility.

Both measures of alpha diversity at two weeks were positively correlated with 30-month negative affect (Shannon: *r* = .35, *p* = .002; Chao1: *r* = .37, *p* < .001), but neither measure of alpha diversity at 18 months was correlated with 30-month negative affect (*ps* > .24). In regression models testing each measure of alpha diversity separately and adjusting for covariates, both Shannon (*β* = 0.40, 95% CI [0.19, 0.60], *p* < .001, *q* = .04) and Chao1 (*β* = 0.36, 95%CI [0.15, 0.58], *p* = .001, *q* = .08) diversity at 2 weeks, but not 18 months (*ps* > .20), positively predicted 30-month negative affect. These results were consistent in regression models with both 2-week and 18-month measures of alpha diversity in them such that higher diversity at 2 weeks, but not 18 months, predicted greater negative affect at 30-months. However, only the association between 2-week Shannon diversity and 30-month negative affect was robust to multiple comparisons correction. These findings are illustrated in Figure 1 and summarized in Table 2.

### Beta diversity analyses

Negative affect was not significantly correlated with Bray-Curtis principal coordinates from either 2 weeks (*ps* > .20) or 18 months (*ps* > .37). Neither 2-week Bray-Curtis principal coordinate predicted 30-month negative affect in a regression model (*ps* > .12). Neither 18-month Bray-Curtis principal coordinate predicted 30-month negative affect in a regression model (*ps* > .22). Beta diversity regression model summaries are in supplementary table 1.

### Differential abundance analyses

#### Hypothesis-driven models

*Bifidobacterium* abundances at 2 weeks (*r* = −.20, *p* = .09) and 18 months (*r* = −.04, *p* = .77) were not significantly correlated with negative affect. *Lactobacillus* abundance at 2 weeks (*r* = .26, *p* = .03), but not 18 months (*r* = −.02, *p* = .85), was correlated with 30-month negative affect. *Bacteroides* abundances from 2 weeks (*r* = .05, *p* = .66) and 18 months (*r* = −.01, *p* = .94) were not correlated with 30-month negative affect. In a series of six regression models, one for each time point for *Bifidobacterium*, *Lactobacillus*, and *Bacteroides*, adjusting for covariates, only 2-week *Lactobacillus* abundance predicted 30-month negative affect, but this association did not survive FDR correction (*β* = 0.26, 95% CI [0.04, 0.48], *p* = .02, *q* = .32). These regressions are summarized in table 3.

#### Exploratory models

In exploratory MaAslin2 models, which perform a linear regression for every genus in the sample against the outcome, no species abundances were significantly associated with 30-month temperament and robust to multiple comparisons correction. At the genus level, 2-week abundances of *Veillonella*, *Streptococcus*, and *Clostridium* were significantly positively associated with negative affect (*Veillonella*: *B* = 1.56, *p* = .02, *q* = .12; *Streptococcus*: *B* = 0.91, *p* = .01, *q* = .12; *Clostridium*: *B* = 1.52, *p* = .05, *q* = .15). In 18-month MaAslin2 models, no species were significantly associated with 30-month negative affect and robust to FDR-correction. At the genus level, abundances of *Anaerotruncus* (*B* = 0.97, *p* = .02, *q* = .24) and *Ruthenibacterium* (*B* = 0.95, *p* = .01, *q* = 0.24) were significantly associated with negative affect. Full microbial abundance MaAslin2 results for each time point can be found in tables S2-S5 in the supplementary materials.

We then performed five regression models, one for each taxon identified in the MaAslin2 models. *Veillonella* abundance from 2 weeks was significantly positively associated with 30-month negative affect when controlling for covariates, but this association was not robust to FDR-correction (*β* = 0.23, 95% CI [0.01, 0.46], *p* = .04, *q* = .32). Two-week *Streptococcus* abundance was positively associated with 30-month negative affect when adjusting for covariates, but this association was not robust to FDR-correction (*β* = 0.28, 95% CI [0.06, 0.50], *p* = .01,*q* = .32). *Clostridium* abundance from 2 weeks was no longer associated with 30-month negative affect when adjusting for covariates (*β* = 0.21, 95% CI [−0.02, 0.44], *p* = .08,*q* = .35). *Anaerotruncus* abundance from 18 months was not significantly associated with 30-month negative affect when adjusting for covariates (*β* = 0.25, 95% CI [−0.002, 0.50], *p* = .05,*q* = .32). *Ruthenibacterium* abundance from 18 months was positively associated with 30-month negative affect when adjusting for covariates, but this association was not robust to FDR-correction (*β* = 0.32, 95% CI [0.07, 0.57], *p* = .02, *q* = .31). Regressions are summarized in table 3.

## Discussion

The current study examined longitudinal associations between the diversity, composition, and functional potential of the gut microbiome at two time points in infancy (2 weeks and 18 months) and a behavioral measure of negative affect at 2.5 years of age. We found that infants who showed higher gut microbiome alpha diversity at 2 weeks, but not 18 months of age, exhibited higher levels of negative affect during a study visit in their home at 30 months of age. We also found some evidence that greater relative abundance of bacteria from the genera *Lactobacillus*, *Streptococcus*, and *Veillonella* when infants were 2 weeks old and *Ruthenibacterium* at 18 months was associated with more toddler negative affect, but these associations did not survive correction for multiple comparisons. These results partially support our preregistered hypothesis that higher infant gut microbiome alpha diversity would be associated with greater negative affect in toddlerhood, and that variation in gut microbiome composition would be associated with variation in toddler negative affect. This study contributes to our growing scientific understanding of early gut-behavior associations with children’s temperament and identifies specific taxa as candidates for further study in the context of affective development.

Our findings that higher alpha diversity at 2 weeks, but not 18 months, was associated with greater negative affect in toddlerhood was partially in line with our preregistered hypotheses and existing literature. We hypothesized that higher alpha diversity across *both* infancy time points would predict more negative affect in toddlerhood. Previous developmental research in humans has found mixed associations between early life alpha diversity and various measures of temperamental risk for psychopathology (Aatsinki et al., 2019; Carlson et al., 2021; Fan et al., 2023; Huovinen et al., 2024; Kelsey et al., 2020; Loughman et al., 2020). The findings reported here partially align with Carlson and colleagues (2021), who found that greater richness (more taxa present) and reduced evenness (dominated by one or a few taxa) at 1 month were associated with greater non-social fear at 12 months, but did not find these associations between 12-month alpha diversity and concurrent fear behavior. Here, we found that higher alpha diversity at two weeks of age, measured by richness and evenness (the Shannon index) was associated with more negative affect at 2.5 years. We did find evidence that higher values of Chao1 diversity, a measure of microbial richness, at two weeks predicted greater negative affect in toddlerhood, but this association did not survive correction for multiple comparisons.

Infants are introduced to a diverse array of microbes from maternal and environmental sources during and immediately following birth. Some of these microbes survive to colonize the infant gut (Ferretti et al., 2018; Penders et al., 2006). Aerobic bacteria, such as Lactobacillaceae, tend to dominate the gut microbiome in the first days of life, followed by a transition to dominance by anaerobic bacteria, namely bifidobacteria and bacteria from the Clostridaceae family (Ahearn-Ford et al., 2022; Penders et al., 2006). Gut microbiome diversity increases dramatically across the first year of life, with marked increases in alpha diversity following the cessation of breastfeeding (Kim et al., 2019; Stewart et al., 2018). As such, higher alpha diversity in infancy may not be developmentally appropriate, and may reflect an accelerated maturation of the infant gut (Beller et al., 2021; Stewart et al., 2018). Previous studies have linked higher alpha diversity in infancy and toddlerhood to prenatal adversity exposure (Querdasi et al., 2023), poorer language outcomes (Carlson et al., 2018), and differences in the development of brain networks implicated in emotional development (Gao et al., 2019). Of note, our values of the Shannon index from 2 weeks and 18 months were not correlated in this sample (*r* = .01, *p* = .90). This suggests that gut microbiome diversity may reflect different ecological states and have different implications for infant health based on developmental timing.

We also identified several taxa of bacteria associated with negative affect, including *Veillonella*, *Lactobacillus*, and *Streptococcus* at 2 weeks of age and *Ruthenibacterium* at 18 months of age, though none of these were robust to false discovery rate correction. These findings are somewhat aligned with previous research finding higher *Veillonella* abundance at 12 months associated with greater concurrent fear behavior (Carlson et al., 2021). Likewise, Aatsinki and colleagues (2019) found positive associations between bacteria from *Veillonella* and infant fear reactivity, in line with our findings, but positive associations between *Streptococcus* and positive affect, in the opposite direction of our findings. We also identified positive associations between 18-month *Ruthenibacterium* abundance and negative affect in toddlerhood. *Ruthenibacterium* has not been as widely studied in relation to the infant gut microbiome, but available evidence has implicated nutrition and feeding interventions in the abundance of *Ruthenibacterium* in early life (Chang et al., 2024; Katagiri et al., 2023), and higher *Ruthenibacterium* abundances have been found in adults with major depressive disorder compared to healthy controls. Not passing FDR correction suggests that the associations we identified between these specific taxa and toddler negative affect have a higher likelihood of being due to random chance, rather than a true effect. When considering the confidence intervals around the coefficient estimates, we generally found that the CIs for many of these taxa had upper or lower bounds very close to zero (*Lactobacillus* had a lower bound of 0.04, *Veillonella* had a lower bound of 0.01), suggesting that the true effect could be zero (or close to zero). Our findings provide support for the idea that these taxa may play an important role in development and should be more closely studied in hypothesis-driven studies of infant neural and behavioral development.

While not robust to FDR correction, we found an unexpected positive association between 2-week *Lactobacillus* abundance and 30-month negative affect. This finding was in the opposite direction of what we had hypothesized. Previous studies in rodents have found that probiotic supplementation with *Lactobacillus* reverses stress-induced changes in behavior in pups (Bravo et al., 2011; Cowan et al., 2016, 2019). In infants, previous studies have found abundances of organisms from *Lactobacillus* in early infancy to be inversely associated with infant crying and fussing (Pärtty et al., 2012), but others have found positive associations between *Lactobacillus* and fear reactivity later in infancy (Carlson et al., 2021). One explanation for these associations is that higher abundance of *Lactobacillus* at two weeks of age might reflect an imbalance of the gut microbiome during the transition from dominance by aerobic microbes, such was *Lactobacillus*, to facultative anaerobes such as *Bifidobacterium*. This would suggest that higher *Lactobacillus* abundance at this point in development might not confer the same advantages that *Lactobacillus* is thought to confer in other developmental periods. Another unexpected finding from these analyses was evidence of inverse associations between socioeconomic status and negative affect that emerged in many of the regression analyses, but not in correlations. While these associations were not all statistically significant, and none survived correction for multiple comparisons, this pattern suggests that SES may explain unique variance in negative affect when other related sources of variance, such as child race or breastfeeding behaviors, are accounted for. Future studies with larger sample sizes will be better situated to examine how SES-related variation in the social environment might impact gut-brain interactions, given documented associations between SES and breastfeeding and other aspects of infant health. Altogether, our findings highlight the importance of considering developmental timing and the broader social context in which development unfolds when interpreting gut microbiome-behavior findings and developing hypotheses for future studies.

Although the body of research examining the mechanisms linking the gut microbiome to behavioral phenotypes is still nascent, emerging evidence suggests the gut microbiome shapes child affect via interactions with the HPA-axis, the vagus nerve, and the immune system—three key pathways of the microbiome-gut-brain axis (Cryan et al., 2019; Yang et al., 2016). Bacteria in the gut produce peptides and other chemicals that interact with vagal afferents in the gastrointestinal tract, which communicate signals from the gut to the brain via the vagus nerve. The vagus nerve, in turn, plays a central role in regulating multiple physiological and neural systems important for behavior, including how the body responds to stress and injury via the HPA-axis and immune system (Biggio et al., 2009; Pavlov & Tracey, 2004). The HPA-axis and vagus nerve have well-documented associations with temperament and emotional development in infancy and early childhood (Blair, 2010; Braren et al., 2021; Shakiba et al., 2023; Wagner et al., 2023). As such, early differences in the composition of the gut microbiome might influence temperament and emotional development by shaping the development of other physiological regulatory systems that support behavior, but future developmental research in humans is needed to test these mechanisms.

The principal limitation of this study is the small sample size, which limits the kind of statistical analyses and conclusions we are powered to draw from our data. Our results should be replicated in a larger sample with more demographic variability and statistical power to detect microbiome/affect associations. Likewise, while our home-based data collection allowed for greater ecological validity in our measurement of infant negative affect, home-based data collection may come at a cost to internal validity. An additional limitation is our lack of an earlier measure of negative affect in this sample, which hinders our ability to examine concurrent associations between the gut microbiome and negative affect. Including earlier measures of negative affect would allow us to disentangle concurrent versus predictive associations between gut microbiome features and temperament. Finally, we lack measures of infant antibiotic use during the first weeks of life, which can have strong effects on the developing infant gut microbiome. Despite these limitations, the study presented here advances the literature in several ways. Longitudinal designs allow us to identify predictive influences of the gut microbiome on child affect, open opportunities for considering prevention of psychopathology, and provide more information about how early differences in the composition and diversity of the gut microbiome influence long-term outcomes. A longitudinal design with multiple time points offers insight into potential timing-specific influences of the gut microbiome on affective development, which has been missing from many previous studies in this area. Finally, the use of whole genome sequencing technology allows for more precision in quantification and classification of microbial abundances. Altogether, these strengths address several existing gaps in the research on infant gut microbiome and temperament. Future longitudinal gut microbiome and child development studies should include larger and more diverse samples, which will help contribute to a broader understanding of microbiome-gut-brain communication across social and cultural contexts and expand the kinds of statistical inferences we can make from data.

The current findings suggest that associations between the gut microbiome and temperament may differ at different points in infancy and suggest the need for further studies characterizing physiological and environmental mechanisms by which the gut microbiome shapes child behavior. Multidisciplinary studies integrating thoughtful measurement of the social environment (including breastfeeding and nutrition practices), high-quality longitudinal gut microbiome data, assessments of the HPA-axis, immune, and autonomic nervous system functioning, and careful multi-method assessment of child behavior will allow us to better understand how early experiences, the gut microbiome, and child behavior together influence risk for psychopathology and begin to uncover some of the physiological mechanisms underlying these associations. Elucidating these complex relationships can help inform interventions to prevent the development of internalizing problems before they arise and promote child physical and mental health throughout the lifespan.

## Supplementary Material

Supplementary materials

**Supplementary material.** To view supplementary material for this article, please visit https://doi.org/10.1017/S0954579425100229.

## Figures and Tables

**Table 1.** Sample characteristics (n = 96)

Variable	N (%) or Mean (SD)
Child sex	45 female (47%)
Child race	
White	60 (63%)
Black	18 (19%)
Hispanic/Latino	10 (10%)
Asian	5 (5%)
Multiple races/other	3 (3%)
Method of delivery	79 vaginal (82%)
Breastfeeding status at 2 weeks (yes/no)	82 yes (85%)
Months of breastfeeding as of 18-month visit	13.14 (6.47)
Primary caregiver education (years)	16.07 (2.26)
Income-to-needs ratio at study enrollment	4.38 (2.52)

**Table 2.** Regression summary of alpha diversity metrics predicting 30-month negative affect

Predictor	*β*	*95%CI*	*p-value*	*q-value*	*R^2^*
Model 1 – 2-week Shannon index (*n* = 71)					.20
**Shannon – 2 weeks**	**0.40**	**[0.19, 0.60]**	< **.001**	**.05**	
Child sex	−0.12	[−0.33, 0.09]	.27	.67	
Child race	0.10	[−0.11, 0.31]	.34	.73	
Family SES	−0.20	[−0.41, 0.02]	.08	.35	
Method of delivery	0.07	[−0.15, 0.28]	.54	.82	
Breastfeeding	0.22	[−0.01, 0.45]	.06	.32	
Model 2 – 2-week Chao1 index (*n* = 71)					.23
Chao1 – 2 weeks	0.36	[0.15, 0.58]	.001	.09	
Child sex	−0.10	[−0.31, 0.12]	.38	.77	
Child race	0.05	[−0.16, 0.27]	.63	.82	
Family SES	−0.14	[−0.36, 0.08]	.21	.65	
Method of delivery	0.08	[−0.14, 0.29]	.47	.82	
Breastfeeding	0.21	[−0.02, 0.44]	.08	.35	
Model 3 – 18-month Shannon index (*n* = 61)					.07
Shannon – 18 months	0.07	[−0.18, 0.32]	.57	.83	
Child sex	−0.02	[−0.28, 0.25]	.91	.96	
Child race	0.08	[−0.17, 0.32]	.55	.82	
Family SES	−0.01	[−0.26, 0.24]	.92	.96	
Method of delivery	0.07	[−0.19, 0.32]	.61	.82	
Breastfeeding	−0.23	[−0.48, 0.02]	.07	.35	
Model 4 – 18-month Chao1 index (*n* = 61)					.09
Chao1 – 18 months	0.16	[−0.09, 0.41]	.20	.65	
Child sex	0.01	[−0.25, 0.27]	.95	.97	
Child race	0.09	[−0.15, 0.33]	.47	.82	
Family SES	0.00	[−0.25, 0.25]	.99	.99	
Method of delivery	0.07	[−0.18, 0.33]	.57	.82	
Breastfeeding	−0.25	[−0.49, −0.01]	.05	.32	
Model 5 – Shannon index, both time points (*n* = 49)					.27
Shannon – 2 weeks	0.33	[0.08, 0.59]	.01	.30	
Shannon – 18 months	0.10	[−0.15, 0.35]	.43	.82	
Child sex	−0.10	[−0.37, 0.18]	.48	.82	
Child race	0.07	[−0.18, 0.32]	.60	.82	
Family SES	−0.24	[−0.49, 0.01]	.05	.31	
Method of delivery	0.09	[−0.18, 0.36]	.51	.82	
Breastfeeding	−0.11	[−0.38, 0.16]	.43	.82	
Model 6 – Chao 1 index, both time points (*n* = 49)					.25
Chao 1 – 2 weeks	0.30	[0.02, 0.57]	0.04	0.32	
Chao 1 – 18 months	0.03	[−0.25, 0.30]	0.85	0.95	
Child sex	−0.03	[−0.32, 0.25]	0.81	0.92	
Child race	0.07	[−0.19, 0.32]	0.61	0.82	
Family SES	−0.18	[−0.44, 0.08]	0.19	0.63	
Method of delivery	0.11	[−0.17, 0.37]	0.45	0.82	
Breastfeeding	−0.18	[−0.44, 0.08]	0.18	0.62	

***Note.* Bold text** indicates significant coefficients in the regression model.

**Table 3.** Regression summary of bacterial genera and negative affect models

Predictor	*β*	95%Confidence Interval	*p-value*	*q-value*	*R^2^*
***Bifidobacterium, 2 weeks* (*n* = 70 )**					.11
Relative abundance	−0.23	[−0.46,0.01]	.06	.32	
Child sex	−0.14	[−0.36, 0.09]	.22	.65	
Child race	0.05	[−0.17, 0.28]	.64	.82	
Family SES	−0.24	[−0.48, −0.01]	.04	.32	
Method of delivery	0.03	[−0.21, 0.27]	.81	.92	
Breastfeeding	0.12	[−0.12, 0.36]	.32	.72	
***Bifidobacterium, 18 months* (*n* = 61)**					.07
Relative abundance	0.02	[−0.25, 0.29]	.88	.96	
Child sex	−0.01	[−0.28, 0.26]	.94	.97	
Child race	0.07	[−0.18, 0.32]	.59	.82	
Family SES	−0.03	[−0.27, 0.22]	.83	.93	
Method of delivery	0.07	[−0.20, 0.34]	.62	.82	
Months of breastfeeding	−0.24	[−0.49, 0.01]	.06	.32	
***Lactobacillus, 2 weeks* (*n* = 70**)					.13
Relative abundance – log transformed	0.26	[0.04, 0.48]	.02	.32	
Child sex	−0.11	[−0.33, 0.12]	.35	.74	
Child race	0.08	[−0.15, 0.29]	.50	.82	
Family SES	−0.14	[−0.37, 0.10]	.26	.67	
Method of delivery	0.10	[−0.12, 0.32]	.38	.77	
Breastfeeding	0.07	[−0.17, 0.31]	.56	.82	
***Lactobacillus, 18 months* (*n* = 61)**					.08
Relative abundance – log transformed	0.13	[−0.11, 0.38]	.29	.68	
Child sex	−0.02	[−0.28, 0.24]	.89	.96	
Child race	0.07	[−0.18, 0.31]	.58	.82	
Family SES	−0.04	[−0.28, 0.21]	.77	.90	
Method of delivery	0.06	[−0.20, 0.31]	.65	.82	
Breastfeeding	−0.26	[−0.51, −0.02]	.04	.32	
***Bacteroides, 2 weeks* (*n* = 70)**					.08
Relative abundance – log transformed	0.10	[−0.13, 0.35]	.37	.77	
Child sex	−0.14	[−0.37, 0.09]	.23	.65	
Child race	0.07	[−0.15, 0.30]	.53	.82	
Family SES	−0.21	[−0.44, 0.03]	.09	.36	
Method of delivery	0.13	[−0.11, 0.37]	.28	.67	
Breastfeeding	0.12	[−0.12, 0.36]	.33	.73	
***Bacteroides, 18 months* (*n* = 61)**					.07
Relative abundance	−0.01	[−0.26, 0.25]	.96	.97	
Child sex	−0.01	[−0.28, 0.25]	.92	.96	
Child race	0.07	[−0.18, 0.32]	.58	.82	
Family SES	−0.03	[−0.28, 0.22]	.81	.92	
Method of delivery	0.06	[−0.20, 0.32]	.63	.82	
Breastfeeding	−0.24	[−0.48, 0.01]	.06	.32	
***Veillonella,* 2 weeks (*n* = 70)**					.12
Relative abundance – log transformed	0.23	[0.01, 0.46]	.04	.32	
Child sex	−0.13	[−0.35, 0.10]	.27	.67	
Child race	0.07	[−0.15, 0.29]	.55	.82	
Family SES	−0.22	[−0.45, 0.01]	.06	.32	
Method of delivery	0.05	[−0.19, 0.28]	.70	.84	
Breastfeeding	0.15	[−0.09, 0.38]	.22	.65	
***Streptococcus, 2 weeks* (*n* = 70)**					.14
Relative abundance – log transformed	0.28	[0.06. 0.50]	.01	.31	
Child sex	−0.11	[−0.33, 0.11]	.32	.72	
Child race	0.08	[−0.14, 0.30]	.48	.82	
Family SES	−0.25	[−0.48, −0.02]	.03	.32	
Method of delivery	0.06	[−0.16, 0.29]	.58	.82	
Breastfeeding	0.14	[−0.09, 0.37]	.23	.65	
***Clostridium, 2 weeks* (*n* = 70)**					.11
Relative abundance – log transformed	0.21	[−0.02, 0.44]	.08	.35	
Child sex	−0.16	[−0.35, 0.10]	.27	.67	
Child race	0.03	[−0.18, 0.26]	.70	.84	
Family SES	−0.27	[−0.46, 0.01]	.06	.32	
Method of delivery	0.03	[−0.15, 0.30]	.52	.82	
Breastfeeding	0.17	[−0.07, 0.42]	.16	.58	
***Anaerotruncus, 18 months* (*n* = 61)**					.12
Relative abundance – log transformed	0.25	[−0.002, 0.50]	.05	.32	
Child sex	0.05	[−0.21, 0.32]	.69	.84	
Child race	0.12	[−0.12, 0.36]	.32	.72	
Family SES	0.01	[−0.23, 0.25]	.93	.96	
Method of delivery	0.02	[−0.24, 0.27]	.90	.96	
Breastfeeding	−0.23	[−0.47, 0.01]	.06	.32	
***Ruthenibacterium, 18 months* (*n* = 61)**					.15
Relative abundance – log transformed	0.32	[0.07, 0.57]	.01	.31	
Child sex	0.06	[−0.20, 0.32]	.66	.82	
Child race	0.18	[−0.07, 0.43]	.16	.58	
Family SES	−0.02	[−0.25, 0.22]	.90	.96	
Method of delivery	0.07	[−0.18, 0.31]	.59	.82	
Breastfeeding	−0.21	[−0.44, 0.03]	.09	.36	
