# Supplementary material for: Longitudinal associations between the infant gut microbiome and negative affect in toddlerhood
Source: Dev Psychopathol. Author manuscript; Available in PMC 2026 Apr 8. (PMC13060658; doi:10.1017/S0954579425100229)
Supplement: Supplementary materials [file NIHMS2153344-supplement-Supplementary_materials.docx]

**Supplementary Materials**

Table S1. Regression summary of beta diversity principal coordinates predicting 30-month negative affect

|  |  | |  | | | | |
| --- | --- | --- | --- | --- | --- | --- | --- |
| **Predictor** | | | **β** | **95% CI** | ***p-value*** | ***q-value*** | ***R^2^*** |
|  | | **2-week Bray-Curtis principal coordinates** | | | |  | 0.10 |
| PCoA1 | | | 0.10 | [-0.14, 0.34] | .43 | .82 |  |
| PCoA2 | | | 0.12 | [-0.05, 0.43] | .12 | .46 |  |
| Child sex | | | -0.15 | [-0.38, 0.08] | .20 | .65 |  |
| Child race | | | .05 | [-0.18, 0.27] | .69 | .84 |  |
| Family SES | | | -0.23 | [-0.47, 0.01] | .06 | .32 |  |
| Breastfeeding | | | 0.13 | [-0.11, 0.37] | .27 | .67 |  |
| Method of delivery | | | .03 | [-0.21, 0.27] | .79 | .92 |  |
|  | | **18-month Bray-Curtis beta diversity** | | | |  | 0.23 |
| PCoA1 | | | -0.05 | [-0.34, 0.24] | .73 | .87 |  |
| PCoA2 | | | 0.17 | [-0.11, 0.45] | .22 | .65 |  |
| Child sex | | | -0.09 | [-0.37, 0.20] | .55 | .82 |  |
| Child race | | | 0.10 | [-0.18, 0.37] | .48 | .82 |  |
| Family SES | | | -0.33 | [-0.59, 0.01] | .01 | .31 |  |
| Breastfeeding | | | -0.26 | [-0.52, 0.01] | .06 | .32 |  |
| Method of delivery | | | 0.07 | [-0.21, 0.36] | .61 | .82 |  |

***Note.* Bold text** indicates significant pathways in the regression model.

Table S2. Full MaAslin2 results for 2-week species abundances predicting 30-month negative affect

| **Species** | **Coefficient** | **Standard Error** | **N** | **N not 0** | **p-value** | **q-value** |
| --- | --- | --- | --- | --- | --- | --- |
| Bifidobacterium breve | -1.3449236 | 0.62476785 | 75 | 70 | 0.03464939 | 0.25584097 |
| Phocaeicola vulgatus | 1.31388123 | 0.6985019 | 75 | 43 | 0.06396024 | 0.25584097 |
| Streptococcus salivarius | 0.63969195 | 0.5325332 | 75 | 67 | 0.23354702 | 0.62279206 |
| Bifidobacterium bifidum | -0.4485234 | 0.70782836 | 75 | 46 | 0.52828234 | 0.75990146 |
| Bifidobacterium longum | -0.1897932 | 0.61870827 | 75 | 72 | 0.75990146 | 0.75990146 |
| Bacteroides fragilis | -0.6012107 | 0.7210702 | 75 | 47 | 0.40712746 | 0.75990146 |
| Staphylococcus epidermidis | 0.30678587 | 0.58399015 | 75 | 44 | 0.60094782 | 0.75990146 |
| Escherichia coli | -0.1597043 | 0.45302477 | 75 | 55 | 0.72545752 | 0.75990146 |

Table S3. Full MaAslin2 results for 2-week genus abundances predicting 30-month negative affect

| **Genus** | **Coefficient** | **Standard Error** | **N** | **N not 0** | **p-value** | **q-value** |
| --- | --- | --- | --- | --- | --- | --- |
| Streptococcus | 0.906607 | 0.354941 | 75 | 74 | 0.012728 | 0.123212 |
| Veillonella | 1.560705 | 0.680208 | 75 | 55 | 0.024642 | 0.123212 |
| Clostridium | 1.51919 | 0.7491 | 75 | 39 | 0.046208 | 0.154027 |
| Bifidobacterium | -0.43365 | 0.628576 | 75 | 74 | 0.492445 | 0.703493 |
| Bacteroides | 0.449109 | 0.567666 | 75 | 69 | 0.43142 | 0.703493 |
| Phocaeicola | 0.594816 | 0.721972 | 75 | 47 | 0.412693 | 0.703493 |
| Enterococcus | 0.585641 | 0.573482 | 75 | 39 | 0.310531 | 0.703493 |
| Staphylococcus | 0.310643 | 0.621206 | 75 | 48 | 0.618534 | 0.773168 |
| Escherichia | -0.13065 | 0.627207 | 75 | 55 | 0.835567 | 0.928407 |
| Klebsiella | 0.019367 | 0.853105 | 75 | 45 | 0.98195 | 0.98195 |

Table S4. Full MaAslin2 results for 18-month species abundances predicting 30-month negative affect

| **Species** | **Coefficient** | **Standard Error** | **N** | **N not 0** | **p-value** | **q-value** |
| --- | --- | --- | --- | --- | --- | --- |
| Bacteroides vulgatus | 1.42370453 | 0.63368771 | 69 | 38 | 0.02795908 | 0.48928391 |
| Ruthenibacterium lactatiformans | 0.92385289 | 0.37321171 | 69 | 58 | 0.01584337 | 0.48928391 |
| Anaerotruncus colihominis | 0.84057162 | 0.41315213 | 69 | 41 | 0.0458586 | 0.53501694 |
| Eubacterium hallii | -0.6538539 | 0.41061795 | 69 | 35 | 0.11600996 | 0.81206972 |
| Faecalibacterium prausnitzii | -0.827592 | 0.48681106 | 69 | 63 | 0.09376405 | 0.81206972 |
| Bifidobacterium longum | -0.560645 | 0.4542966 | 69 | 61 | 0.22147899 | 0.9401247 |
| Eubacterium eligens | -0.4149327 | 0.34802714 | 69 | 42 | 0.23737189 | 0.9401247 |
| Ruminococcus gnavus | 0.42498994 | 0.36600144 | 69 | 64 | 0.24969303 | 0.9401247 |
| Agathobaculum butyriciproducens | -0.4924112 | 0.35859142 | 69 | 39 | 0.17427407 | 0.9401247 |
| Escherichia coli | 0.50893186 | 0.45622287 | 69 | 40 | 0.26860706 | 0.9401247 |
| Bifidobacterium breve | -0.3607847 | 0.62302051 | 69 | 39 | 0.56447062 | 0.95894428 |
| Bifidobacterium pseudocatenulatum | -0.6428129 | 0.65195608 | 69 | 39 | 0.32769233 | 0.95894428 |
| Eggerthella lenta | 0.02604416 | 0.21545377 | 69 | 67 | 0.90414747 | 0.95894428 |
| Gordonibacter pamelaeae | 0.04737959 | 0.28352549 | 69 | 67 | 0.86778781 | 0.95894428 |
| Bacteroides fragilis | -0.1269551 | 0.56008272 | 69 | 46 | 0.82136895 | 0.95894428 |
| Bacteroides uniformis | 0.23753227 | 0.79597575 | 69 | 43 | 0.76630887 | 0.95894428 |
| Streptococcus salivarius | 0.39416261 | 0.40231927 | 69 | 60 | 0.33074595 | 0.95894428 |
| Hungatella hathewayi | 0.22581572 | 0.42710631 | 69 | 55 | 0.59875324 | 0.95894428 |
| Intestinimonas butyriciproducens | -0.4193897 | 0.50206409 | 69 | 39 | 0.40650036 | 0.95894428 |
| Blautia wexlerae | 0.05729865 | 0.34625604 | 69 | 62 | 0.86906403 | 0.95894428 |
| Fusicatenibacter saccharivorans | -0.0558321 | 0.36622316 | 69 | 43 | 0.87928723 | 0.95894428 |
| Clostridium bolteae | 0.24121343 | 0.38543055 | 69 | 56 | 0.5335536 | 0.95894428 |
| Clostridium symbiosum | 0.30133162 | 0.37160351 | 69 | 53 | 0.42029687 | 0.95894428 |
| Eubacterium rectale | 0.06804611 | 0.48477683 | 69 | 43 | 0.88879208 | 0.95894428 |
| Roseburia faecis | 0.06114254 | 0.32386747 | 69 | 42 | 0.85082907 | 0.95894428 |
| Roseburia intestinalis | 0.40657612 | 0.56582017 | 69 | 42 | 0.47491015 | 0.95894428 |
| Roseburia inulinivorans | -0.2396565 | 0.37687889 | 69 | 38 | 0.52700923 | 0.95894428 |
| Sellimonas intestinalis | -0.1020273 | 0.47538449 | 69 | 43 | 0.83071501 | 0.95894428 |
| Flavonifractor plautii | 0.05888901 | 0.22308674 | 69 | 68 | 0.79261051 | 0.95894428 |
| Ruminococcus bromii | 0.24250845 | 0.34090966 | 69 | 37 | 0.47933246 | 0.95894428 |
| Clostridium innocuum | -0.1088905 | 0.45656253 | 69 | 45 | 0.81222067 | 0.95894428 |
| Dialister invisus | 0.36432131 | 0.40923878 | 69 | 40 | 0.37652185 | 0.95894428 |
| Proteobacteria bacterium CAG 139 | -0.2303247 | 0.5229686 | 69 | 35 | 0.66105212 | 0.95894428 |
| Anaerostipes hadrus | -0.0038371 | 0.30789562 | 69 | 59 | 0.9900937 | 0.9900937 |
| Parasutterella excrementihominis | -0.0110643 | 0.46149588 | 69 | 35 | 0.98094394 | 0.9900937 |

Table S5. Full MaAslin2 results for 18-month genus abundances predicting 30-month negative affect

| **Genus** | **Coefficient** | **Standard Error** | **N** | **N not 0** | **p-value** | **q-value** |
| --- | --- | --- | --- | --- | --- | --- |
| Anaerotruncus | 0.968996 | 0.391346 | 69 | 42 | 0.015817 | 0.237257 |
| Ruthenibacterium | 0.953596 | 0.358693 | 69 | 58 | 0.009806 | 0.237257 |
| Bifidobacterium | -0.33103 | 0.229906 | 69 | 66 | 0.154565 | 0.597911 |
| Clostridium | 0.702573 | 0.491816 | 69 | 54 | 0.157785 | 0.597911 |
| Eisenbergiella | 0.642137 | 0.357206 | 69 | 40 | 0.076736 | 0.597911 |
| Agathobaculum | -0.5472 | 0.384603 | 69 | 39 | 0.159443 | 0.597911 |
| Faecalibacterium | -0.79598 | 0.4943 | 69 | 63 | 0.112029 | 0.597911 |
| Veillonella | 0.62529 | 0.423868 | 69 | 38 | 0.144843 | 0.597911 |
| Escherichia | 0.545313 | 0.458908 | 69 | 40 | 0.238916 | 0.796387 |
| Lachnoclostridium | 0.280364 | 0.27519 | 69 | 66 | 0.311961 | 0.850803 |
| Dialister | 0.403235 | 0.393301 | 69 | 40 | 0.308929 | 0.850803 |
| Collinsella | -0.09791 | 0.674941 | 69 | 41 | 0.885091 | 0.979861 |
| Eggerthella | 0.05401 | 0.218523 | 69 | 67 | 0.80554 | 0.979861 |
| Gordonibacter | 0.084692 | 0.275438 | 69 | 67 | 0.75943 | 0.979861 |
| Bacteroides | -0.22589 | 0.348718 | 69 | 67 | 0.519338 | 0.979861 |
| Streptococcus | 0.213441 | 0.391136 | 69 | 63 | 0.587088 | 0.979861 |
| Hungatella | 0.25881 | 0.413549 | 69 | 55 | 0.533555 | 0.979861 |
| Intestinimonas | -0.40362 | 0.496571 | 69 | 39 | 0.419203 | 0.979861 |
| Eubacterium | -0.30375 | 0.405405 | 69 | 57 | 0.456336 | 0.979861 |
| Anaerostipes | -0.05331 | 0.324018 | 69 | 60 | 0.869815 | 0.979861 |
| Blautia | 0.017986 | 0.187013 | 69 | 68 | 0.923668 | 0.979861 |
| Fusicatenibacter | -0.04488 | 0.360443 | 69 | 43 | 0.90128 | 0.979861 |
| Lachnospiraceae (unclassified) | 0.05884 | 0.50962 | 69 | 43 | 0.908427 | 0.979861 |
| Roseburia | -0.1936 | 0.377202 | 69 | 61 | 0.609463 | 0.979861 |
| Sellimonas | -0.09348 | 0.504483 | 69 | 43 | 0.853548 | 0.979861 |
| Flavonifractor | 0.10279 | 0.205234 | 69 | 68 | 0.618123 | 0.979861 |
| Ruminococcus | -0.17458 | 0.61815 | 69 | 52 | 0.778494 | 0.979861 |
| Erysipelatoclostridium | -0.01154 | 0.455468 | 69 | 53 | 0.979861 | 0.979861 |
| Parasutterella | 0.019133 | 0.437756 | 69 | 35 | 0.965268 | 0.979861 |
| Proteobacteria (unclassified) | -0.19711 | 0.495462 | 69 | 35 | 0.692014 | 0.979861 |
